# Tocilizumab in HIV patient with severe COVID-19: case report

**DOI:** 10.1186/s12981-021-00404-5

**Published:** 2021-10-16

**Authors:** Pedro Vieira Bertozzi, Amanda de Oliveira Vicente, Amanda Siqueira Pereira, Joao Pedro Espinha de Sant’Ana, Rafaela Braga Cabrera Mano, Luciana Souza Jorge, Rodrigo Afonso da Silva Sardenberg

**Affiliations:** 1 Advanced Research Center in Medicine, Uniao das Faculdades dos Grandes Lagos, UNILAGO, São Jose do Rio Preto, SP Brazil; 2 Infectology Department, Hospital de Base, São Jose do Rio Preto, Brazil; 3 Thoracic Surgery Department, Hospital Alemao Oswaldo Cruz, Sao Paulo, Brazil; 4 Advanced Research Center in Medicine, Uniao das Faculdades dos Grandes Lagos—UNILAGO, Emilia Tarraf st., 340, São Jose do Rio Preto, SP 15055-460 Brazil

**Keywords:** COVID-19, HIV, Tocilizumab, Coinfection, Case report

## Abstract

**Background:**

A 73-year-old male patient who had a history of Human Immunodeficiency Virus (HIV) infection for over 20 years was diagnosed with SARS-CoV-2 infection.

**Case presentation:**

The patient was admitted to the Intensive Care Unit (ICU), where he remained for 25 days, due to a severe condition. Intubation, hemodialysis, and tracheostomy were necessary to maintain homeostasis. In addition to regular treatment with etravirine, dolutegravir, darunavir, and ritonavir for highly active antiretroviral therapy, the patient received tocilizumab, which showed a great recovery in the patient’s condition.

**Conclusion:**

The patient had several risk factors, such as male gender, age  >  70 years, and arterial hypertension. The use of tocilizumab was of great importance in the patient’s recovery since the drug increased his immune response, which is deficient, due to HIV infection.

## Background

A 73-year-old patient with a history of Human Immunodeficiency Virus (HIV) infection for more than 20 years was diagnosed with SARS-CoV-2 infection. The patient was admitted to the ICU, where he remained for 25 days. In addition to regular treatment, the patient received tocilizumab, showing excellent recovery from severe symptoms.

Thus, we aim to show how important the implementation of the immunosuppressant was to help in the immune response against COVID-19, combined with the non-interruption of the highly active antiretroviral therapy.

## Introduction

Since December 2019, we have faced a pandemic of COVID-19, which had its first outbreak in Wuhan, China. The etiological agent was identified as a new Coronavirus closely related to the former SARS-CoV epidemic, therefore named SARS-CoV-2, a medium-sized envelope enveloped RNA virus. The disease, COVID-19, was recognized as a pandemic by the World Health Organization (WHO) in March 2020. As of the date of publication of this report, more than 2,750,000 deaths have been confirmed due to COVID-19 [1, 2]. COVID-19 presents a risk to people living with HIV (PLHIV), particularly in those with low TCD4  +  cell counts, high viral load and other comorbidities. Recent studies suggest that infection rates in HIV carriers are equal to those in the general population, but there may be a correlation between low CD4 levels and disease severity [3, 4].

In the present report, we describe the case of a 73-year-old HIV-positive man who successfully evolved from severe acute respiratory syndrome.

## Case presentation

On March 21, a 73-year-old man entered the emergency room complaining of fever and dyspnea progressing over 10 days. Physical examination showed axillary temperature  =  38.6 °C, BP (blood pressure)  =  103/73 mmHg, heart rate  =  88 bpm, respiratory rate  =  39 bpm and Sat 89% (O_2_ nasal cannula with 3 L/min). He was put under a non-rebreather mask with 10 L/min and was admitted to the ICU.

He had a history of HIV infection for over 20 years, and at the time of the SARS-CoV-2 infection the patient was under treatment regular with etravirine (200 mg bid), dolutegravir (50 mg bid), darunavir (600 mg bid), and ritonavir (100 mg od) for antiretroviral therapy (ART) due to virological failure. The last TCD4 + cell count was above 500/µL and the viral load was undetectable for eight years. He also had a previous diagnosis of systemic arterial hypertension (SAH), which was controlled using captopril (25 mg od), and furosemide (40 mg od). The patient has no history of diabetes mellitus or smoking.

On the day of admission (March 21), a nasopharyngeal swab was collected, and the Real-Time Polymerase Chain Reaction (RT-PCR) for SARS-CoV-2 was positive. The virus panel for Adenovirus, Coronavirus types 229E, HKU1, NL63 and OC43, Enterovirus/Rhinovirus, Influenza types A, A/H1 and B, human Metapneumovirus, Parainfluenza types 1, 2, 3 and 4, Respiratory Syncytial Virus, *Bordetella pertussis*, *Chlamydophila pneumoniae,* and *Mycoplasma pneumoniae* were negative.

**Fig. 1:**
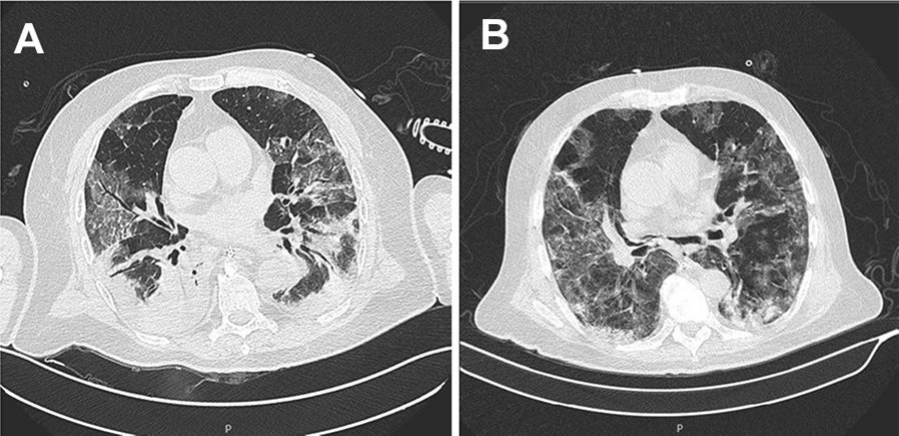
**A** CT on the admission day. CT showing ground glass opacity (GGO) areas with the presence of consolidation areas in the posterior region. **B** CT after 22 days of hospitalization. CT with replacement of the consolidation for ground glass opacity (GGO) areas, demonstrating significant improvement of the clinical condition

A chest computed tomography (CT) scan on admission (Fig. 1A) showed multiple ground-glass opacities, affecting all pulmonary lobes with predominantly peripheral distribution, associated with consolidation on lower lobes, with accented pulmonary involvement (>  50%). Empirical broad-spectrum antimicrobial therapy with ceftriaxone (2 g od) and azithromycin (500 mg od) was started, the ART was maintained, and enoxaparin was prescribed (40 mg od).

The laboratorial exams revealed high percentage of white blood cell, a normal neutrophil count 6410/mm^3^ (normal range: 1700–7000/mm^3^), a normal percentage lymphocyte count 1050/mm^3^ (normal range: 900–2900/mm^3^). There was creatinine (CR) level was 2.6 mg/dL (normal range: 0.7–1.3 mg/dL) elevated blood levels of C-reactive protein (CRP) 34.17 mg/L (normal range: 0–5 mg/L), D-dimer 8.469 ng/mL (normal range:  <  0.5 ng/mL), and Lactic Dehydrogenase (LDH) 592 U/L (normal range: 240–480 U/L). The viral load was undetectable, and the CD4 cell count was 876 cell/mm^3^ (normal range: 404–1612 cell/mm^3^) and CD8 cell count was 876 cell/mm^3^ (normal range: 229–1129 cell/mm^3^).

On March 22, due to need for progressive O2 volume, Sat 86%, respiratory distress despite noninvasive ventilation, and tachypneia, the patient underwent endotracheal intubation and invasive mechanical ventilation in protective-ventilation strategy (Tidal Volume of 6–8 ml/kg), FIO2 60%, PEEP 8 mmHg (to reduce driving pressure levels and therefore lung injury) and pressure support 20 mmHg (spontaneous ventilation mode with limited plateau pressure decreasing chances of barotrauma). Blood gas analysis revealed pH 7.32 (normal range: 7.35–7.45), PaO2 116 mmHg (normal range:  >  60 mmHg), PaCO2 38 mmHg (normal range: 35–45 mmHg) and SatO_2_ 98% (normal range: 94–100%). Ceftriaxone and azithromycin were prescribed for empiric therapy for bacterial besides hydroxychloroquine (HCQ) (100 mg od). In addition to previous use of Etravirin, Dolutegravir, Darunavir, and Ritonavir for HIV maintenance treatment.

On March 28, the use of Azithromycin and HCQ was interrupted. The central venous catheter blood cultures identified *Staphylococcus epidermidis* and *Staphylococcus hominis* resistant to methicillin and sensibility only to linezolid. On the same day, *Candida dubliniensis* was isolated in the tracheal secretion and therefore Meropenem, Linezolid, and Micafungin were incorporated into the treatment due to severely critical illness. Laboratory test revealed a worsening renal disfunction (CR 4.2 mg/dL) and he presented low diuresis (700 mL/24 h), thus continuous venovenous hemodialysis (CVVHD) was initiated.

On April 2nd, the interleukin-6 (IL-6) measurement was 79.3 pg/mL (reference value: 1.5–7 pg/mL). The patient received tocilizumab 8 mg/kg (600 mg) intravenously. One day after, the patient gets defervescence for the first time since he was admitted, and started to improve progressively.

On April 7, tracheostomy was performed, and five days later, he presented an improvement in respiratory condition—CT done on April 15 showed a significant improvement in pulmonary involvement (Fig. 1B), and mechanical ventilation was no longer needed.

**Fig. 2:**
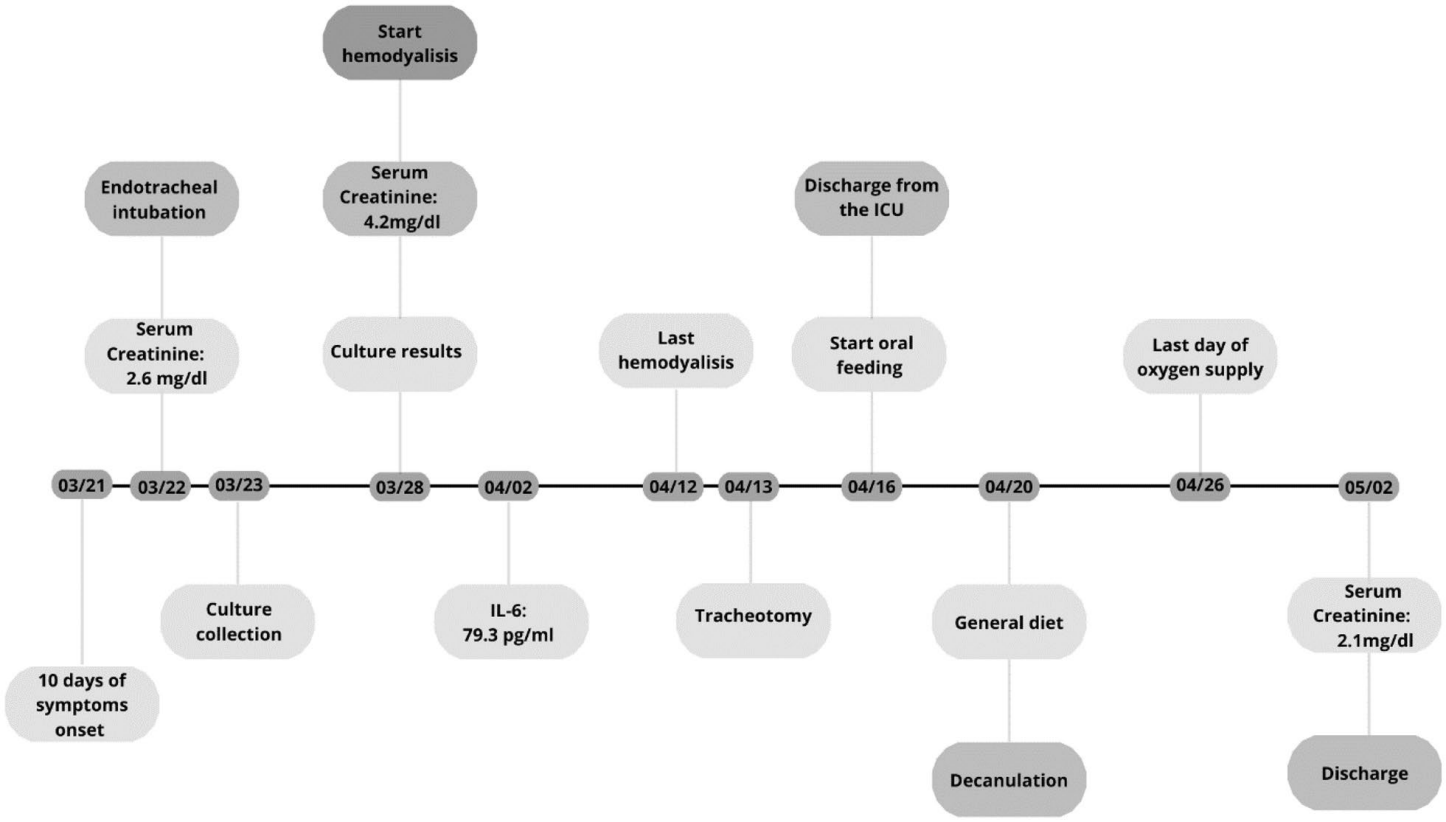
Patient’s clinical evolution and procedures over time

On April 15, the patient was discharged from the ICU and seven days later, the tracheostomy was removed. On May 2nd, the patient was discharged from the hospital, and no prescription was needed unless the use of his regular medications. The clinical chronologic outcome is showed on Fig. 2.

## Discussion

Worldwide, there are approximately 38 million people living with HIV, and under a higher risk of being exposed to SARS-CoV-2 [3]. In Brazil, 9,66,058 people have been infected since 1980, being 51.2% in the Southeastern area, where the city of São Paulo is located [5]. According to the Centers for Disease Control and Prevention (CDC), people with HIV are in the COVID-19 risk group, needing extra precautions. It also affirms that people with a low CD4 cell count and those who are not on HIV treatment (ART) are under an even higher probability of getting sick [6].

The HIV physiopathology consists of destroying the immune cells system and specific antibody responses, which in cases of COVID-19 association may cause a longer course of the disease [7]. A recent study has shown that acute respiratory infections increased mortality in HIV-positive people in Africa [1]. A cohort study in South Africa showed that PLWH should be considered high-risk group for COVID-19 management, especially if they have other comorbidities. A great amount of PLWH who died from COVID-19 also had diabetes (50%) and hypertension (42%) [8]. Nevertheless, studies from other countries presented a different outcome, showing that COVID-19 and PLHIV association didn’t impact the survival [9].

A recent New York State (NYS) retrospective study showed that infection and mortality rates among PLWH did not present a significant difference between the rate of general population. The exception is among the over 40 years old patients, which rates are significantly higher. According to the same study, PLWH have high rates of hospitalization, 8.29 per 1000 versus 3.15 per 1000 in the general population [10]. The ISARIC WHO CCP-UK study found similar ratios of admission to critical care between PLWH and HIV-negative people. As for the mortality rates, after using an age-adjusted statistical analyses concluded that PLWH hospitalized with COVID-19 had increased risk of day-28 mortality [11].

The fact that the patient was already taking antiretroviral medication is a hypothesis that would explain this scenario [9]. Another factor that seems to have a positive impact on the outcome of the patients is the undetectable viral load, as recent studies suggest it results in a better general prognosis [12].

Generalized immune activation and systemic CD4 lymphocyte depletion occur in the chronic phase of untreated HIV; also, the remaining T cells may initiate abnormal responses to antigens. The lymphocyte B dysfunction results in abnormal polyclonal activation and a lack of specific antibody responses. COVID-19 causes a significant reduction in the total number of lymphocyte B, lymphocyte T and natural killer (NK) cells. The pathophysiology of the severe cases is not yet fully understood, but it appears not only to be related to virulence factors of the pathogen, but also to the dysfunctional and self-aggressive immune response triggered by the infection. This phenomenon has been called “cytokine storm” and usually occurs in the second week of infection for COVID-19, which was observed in the patient in this brief report, admitted on the tenth day of symptoms [13].

A recent study suggests that SARS-CoV-2 may damage lymphocytes, especially T lymphocytes, and the immune system was harm during the period of the disease [7]. In addition to these findings, a characteristic of COVID-19 patients is an increased laboratory level of CRP, D-dimer, and LDH, as our patient presented on the admission exams, the lung is the main organ affected by this virus, which will cause microvascular lesions, due to a systemic inflammatory response (SIR). The intestines and kidneys also have receptors that are affected by SIR, but the main concern is with the kidneys, which by this process can generate low diuresis, proteinuria, and changes in the amounts of albumin in the urine, requiring hemodialysis [9, 14]. At this case, we can’t justifiy the previous renal injury (high serum creatinine level at admission) due to HIV nephropathy or by ART toxicity.

The patient’s condition (a 60-year-old man, presenting immunodeficiency, characterized by decreased in immunological system functions) triggers a rise in the incidence and severity of infectious diseases, explaining the risk of COVID-19 in this group [15]. Besides being elderly, the patient has a history of SAH. When this chronic disease is treated with ACE inhibitors, the present upregulation of the ACE2 receptor and the raise expression of ACE2 could facilitate the viral entry [16]. Beyond that, the heart and lungs present a high expression of ACE2 receptors [17].

Pulmonary involvement of COVID-19 was initially treated with ceftriaxone to bacterial coinfection and HCQ since preliminary in vitro studies on viral infection models showed potential antiviral activities, chloroquine (CQ)/hydroxychloroquine (HCQ), and azithromycin; however, the clinical studies on COVID-19 patients treated with CQ/HCQ and azithromycin led to controversies in different regions due to their adverse side effects [18].

The empiric therapy for polymicrobial infection was replaced by meropenem, linezolid and micafungin, despite blood and tracheal secretion cultures result in colonizing bacteria due to the worsening of the patient’s pulmonary clinical condition. Although Vancomycin is a high blood concentration drug, Linezolid was the drug of choice for treatment of coagulase-negative-staphylococci, because it presents less nephrotoxicity in a severely critically patient with previous renal injury. Linezolid also has the advantage of its high concentration in lung tissue [19, 20]. Daptomycin was not the drug of choice because it does not belong to the protocol of drugs used in the hospital in question.

The higher levels of interleukin-6 have been also correlated with the severe cases of COVID-19. Tocilizumab, an anti-interleukin-6 receptor monoclonal antibody, is used in the treatment of various inflammatory conditions. A recent study showed that the use of tocilizumab in hospitalized patients with COVID-19 demonstrated a lower percentage of patients that required mechanical ventilation when compared to the group that did not receive it. Even though this study presents the same survival rates when including deaths from any cause, the use of tocilizumab might be beneficial to some patients, especially moderate or severe cases that have not yet received mechanical ventilation. In this case, tocilizumab was introduced in the patient’s treatment after the patient underwent mechanical ventilation, but yet presented an improvement in the clinical condition [21].

Regarding the treatment, the use of tocilizumab, ART, and broad-spectrum antibiotics is in line with the most recent literature for the treatment of COVID-19 in patients with impaired immune system functions. The only medication that has not been administered is Dexamethasone, which has an important reducing factor in lung injuries and was demonstrated to have better results regarding mortality rate in patients with moderate and severe COVID-19. However, at the time of the patient's acquisition of severe COVID-19 [22, 23], the main studies on the impact of Dexamethasone on COVID-19 mortality had not been published. Therefore, the patient did not receive corticotherapy during hospitalization.

## Conclusion

The reported patient presents the following variables as risk factors: male sex, age  >  70 years, and hypertension. Despite HIV infection, the patient had good adherence to ART, TCD4 cells  >  500/µL, and long-standing undetectable viral load. We chose to maintain ART throughout the hospitalization and this decision may have contributed to the good outcome. It is possible that all the severity of the case was due to infection by SARS-CoV-2 and a dysfunctional immune response.

## Data Availability

All data generated or analysed during this study are included in this published article.

## Abbreviations

HIV: Human Immunodefiency Virus
ICU: Intensive Care Unit
PLHIV: People living with HIV
ART: Antiretroviral therapy
SAH: Systemic arterial hypertension
CT: Computed tomography
CR: Creatinine
CRP: C-reactive protein
LDH: Lactic dehydrogenase
HCQ: Hydroxychloroquine
CVVHD: Continuos veno-venous hemodialysis
NK: Natural killer
SIR: Systemic inflammatory response
